# Assessment of genetic diversity and variety identification based on developed retrotransposon-based insertion polymorphism (RBIP) markers in sweet potato (*Ipomoea batatas* (L.) Lam.)

**DOI:** 10.1038/s41598-021-95876-w

**Published:** 2021-08-24

**Authors:** Yusha Meng, Wenjin Su, Yanping Ma, Lei Liu, Xingguo Gu, Dianxing Wu, Xiaoli Shu, Qixian Lai, Yong Tang, Liehong Wu, Yin Wang

**Affiliations:** 1 Institute of Rural Development, Zhejiang Academy of Agricultural Sciences, Hangzhou, 310021 People’s Republic of China; 2 Key Laboratory of Creative Agriculture, Ministry of Agriculture, Hangzhou, 310021 People’s Republic of China; 3 Institute of Food Crops, Hubei Academy of Agricultural Sciences, Wuhan, 430064 People’s Republic of China; 4 State Key Laboratory of Rice Biology, Institute of Nuclear Agriculture Sciences, Zhejiang University, Hangzhou, 310029 People’s Republic of China

**Keywords:** Plant sciences, Plant breeding, Plant genetics

## Abstract

Sweet potato, a dicotyledonous and perennial plant, is the third tuber/root crop species behind potato and cassava in terms of production. Long terminal repeat (LTR) retrotransposons are highly abundant in sweet potato, contributing to genetic diversity. Retrotransposon-based insertion polymorphism (RBIP) is a high-throughput marker system to study the genetic diversity of plant species. To date, there have been no transposon marker-based genetic diversity analyses of sweet potato. Here, we reported a structure-based analysis of the sweet potato genome, a total of 21555 LTR retrotransposons, which belonged to the main LTR-retrotransposon subfamilies *Ty3-gypsy* and *Ty1-copia* were identified. After searching and selecting using Hidden Markov Models (HMMs), 1616 LTR retrotransposon sequences containing at least two models were screened. A total of 48 RBIP primers were synthesized based on the high copy numbers of conserved LTR sequences. Fifty-six amplicons with an average polymorphism of 91.07% were generated in 105 sweet potato germplasm resources based on RBIP markers. A Unweighted Pair Group Method with Arithmatic Mean (UPGMA) dendrogram, a model-based genetic structure and principal component analysis divided the sweet potato germplasms into 3 groups containing 8, 53, and 44 germplasms. All the three analyses produced significant groupwise consensus. However, almost all the germplasms contained only one primary locus. The analysis of molecular variance (AMOVA) among the groups indicated higher intergroup genetic variation (53%) than intrapopulation genetic variation. In addition, long-term self-retention may cause some germplasm resources to exhibit variable segregation. These results suggest that these sweet potato germplasms are not well evolutionarily diversified, although geographic speciation could have occurred at a limited level. This study highlights the utility of RBIP markers for determining the intraspecies variability of sweet potato and have the potential to be used as core primer pairs for variety identification, genetic diversity assessment and linkage map construction. The results could provide a good theoretical reference and guidance for germplasm research and breeding.

## Introduction

Sweet potato (*Ipomoea batatas* (L.) Lam.) is regarded as the world’s seventh most important food crop and can be used as a staple food, animal feed, industrial raw material to extract starch as well as in alcohol and biofuel. In addition, orange-fleshed sweet potato has a high level of β-carotene, which could be used to prevent vitamin A deficiency-related blindness and maternal mortality in many developing countries. Due to its high productivity and adaptability to a wide range of environmental conditions, sweet potato is cultivated in more than 100 countries worldwide, particularly in the developing countries of Sub-Saharan Africa and South Asia. China is the largest producer of sweet potato, where several cultivars have been developed over 100 years of cultivation. However, information regarding the genetic diversity of Chinese sweet potato germplasm remains limited due to the complicated genome of this species, which limits the process of developing improved cultivars^1,2^. To establish effective breeding strategies, it is necessary to analyze the genetic diversity, evaluate the genetic structure and understand the genetic background among sweet potato accessions.

In recent years, several morphological and molecular markers have been developed to assess the genetic diversity of sweet potato germplasm, including random amplified polymorphic DNAs (RAPDs)^3–5^, amplified fragment length polymorphisms (AFLPs)^6,7^ (Li et al., 2009; Liu et al., 2012), simple sequence repeats (SSRs)^8–10^, and single nucleotide polymorphisms (SNPs)^2^. However, for the massive genome sequences of sweet potato, these published markers are not sufficient to construct a high-density genetic map that could be highly useful for genetic studies. Thus, there is a great need for the exploration of new molecular markers.

Repetitive sequences make up a large proportion of the plant genome. Among repetitive sequences are transposable elements (TEs), which are grouped into two main classes according to their transposition intermediate^11^. Retrotransposons are a widespread class of TEs that exist in all plant species investigated to date^11^. Long terminal repeat (LTR) retrotransposons are one of the most important transposon families^12,13^. LTRs are easy to find because of their presence as flanking sequences at the 5’ and 3’ ends of coding regions in the genome^14^. Based on the above characteristics and their ubiquitous distribution, abundant copy number and insertion polymorphisms, LTRs are valuable for developing new molecular markers^15,16^. Compared with the traditional molecular markers mentioned above, retrotransposon-based markers have advantages including abundant polymorphisms and good reproducibility and genome coverage. Recently, several types of retrotransposon-based DNA markers have been developed and widely applied in evaluating genetic diversity and constructing linkage maps of numerous plant species^1,17–24^. These studies have confirmed that retrotransposon-based DNA markers are suitable for genetic diversity analysis. Unfortunately, there are few reports on the application of retrotransposon-based DNA markers in assessing the genetic diversity of sweet potato.

In this research, we report the development of new retrotransposon-based insertion polymorphism markers (RBIPs) derived from the genome sequence of the sweet potato cultivar Taizhong No. 6 (China national accession number 2013003) and evaluated the capacity and efficiency of these markers for distinguishing genetic diversity in 105 cultivars. The primary objective of this work is to provide new insights into the classification of sweet potato and to assist in the genetic research and breeding of sweet potato.

## Results

### Discovery and classification of LTR retrotransposons in the sweet potato genome

A total of 21555 LTR retrotransposons were obtained, making up 1.1% of the sweet potato genome^25^ (870 Mb). According to the sequence similarity with the reported retrotransposons, 13002 LTR retrotransposons were assigned to the *copia* family, 8114 LTR retrotransposons belonged to the *gypsy* family, and 439 LTR retrotransposons were classified to other families. The *copia* retrotransposons were further clustered into 12342 subfamilies with a single LTR retrotransposon sequence, 23 subfamilies with two LTR retrotransposon sequences, and 85 subfamilies with three or more LTR retrotransposon sequences (Table 1). The *gypsy* retrotransposons were clustered into 7775 subfamilies with a single LTR retrotransposon sequence, 37 subfamilies with two LTR retrotransposon sequences, and 49 subfamilies with three or more LTR retrotransposon sequences (Table 1). After searching HMMs in the 21555 LTR retrotransposons, 1616 LTR-RT sequences containing at least two models were screened and used for subsequent analysis. The 1616 LTR-RT sequences included 1311 *copia* families and 305 *gypsy* families.

**Table 1:** Classification of LTRs in different families and subfamilies.

Subfamily cluster number	*Copia* family		*Gypsy* family	
	Subfamily number	LTR-RT number	Subfamily number	LTR-RT number
1	12,342	12,342	7775	7775
2	23	46	37	74
> = 3	85	614	49	265
Total	12,450	13,002	7861	8114

### Development and evaluation of RBIP primers

According to the principle of primer design, 48 pairs of RBIP primers were finally developed from the 1616 LTR-RT sequences, 6 pairs were from the *copia* 1 subfamily, 15 pairs were from the *copia* 2 subfamily, and 27 pairs were from the *gypsy* 2 subfamily (Supplementary Table 1). The Tm values of the RBIP primers ranged from 51.1 to 59.12 °C, and the GC content ranged from 35 to 55%. The length of the amplified products was 152–993 bp, with an average of 456 bp (Supplementary Table 1).

The 48 RBIP primers were evaluated in 105 sweet potato germplasm resources (Table 2) and generated 64 marker candidates (23, no amplification; 6, monomorphism; 13, unstable amplification among resources) (Supplementary Figure 1). The remaining 6 (12.5%) pairs of primers (4 from the *copia* 2 subfamily, 2 from the *gypsy* 2 subfamily) showed clear and stable amplified fragments with polymorphisms among the 105 resources.

**Table 2:** The 105 sweet potato germplasms used in this study and their origins.

Number	Name	Origin	Number	Name	Origin
1	Jinqing	Xiaoshan District	54	Xiaoshanjinqing	Xiaoshan District
2	Linhai	Linhai City	55	Hongpibaixin-6	Chun'an County
3	Huyuan	Jinyun County	56	Datoubai	Cangnan County
4	Chaoshu	Cangnan County	57	Xinhong 3	Cangnan County
5	Zitong 1	Chun’an County	58	Liusiguang	Jinyun County
6	Zhe 81	Zhejiang Academy of Agricultural Sciences	59	Xiaoshanlouta	Xiaoshan District
7	Liushiri-1	Fuyang District	60	Lianhuaru	Cangnan County
8	Hongpibaixin-1	Wuyi County	61	Zhe 38	Zhejiang Academy of Agricultural Sciences
9	Shenglibaihao-1	Chengzhou City	62	Guangshu 87	Guangdong Academy of Agricultural Sciences
10	Xushu 18–1	Chengzhou City	63	Zheshu 2	Zhejiang Academy of Agricultural Sciences
11	Nanjingzhong	Yongkang City	64	Zheshu 77	Zhejiang Academy of Agricultural Sciences
12	Chaosheng 5	Chengzhou City	65	Zhe 255	Zhejiang Academy of Agricultural Sciences
13	Jiande	Jiande City	66	Shenglibaihao-5	Tongxiang City
14	Jizhuafanshu	Chun'an County	67	Hongpibaixin-7	Dongyang City
15	Anyangbaifanshu	Chun'an County	68	Zheshu 48	Zhejiang Academy of Agricultural Sciences
16	Hangzhoufanshu	Pujiang County	69	Midong	Cangnan County
17	Shenglibaihao-2	Linhai City	70	Hongpibaixin-8	Jiande City
18	Xinzhonghua-1	Suichang County	71	Mei 1	America
19	Hongpibaixin-2	Ninghai County	72	Zheshu 6025	Zhejiang Academy of Agricultural Sciences
20	Hongpihuangxin-1	Dongyang City	73	Xinxiang	Zhejiang Academy of Agricultural Sciences
21	Jinguahuangfanshu	Yongkang City	74	Zhezishu 5	Zhejiang Academy of Agricultural Sciences
22	Baishu	Zhejiang Academy of Agricultural Sciences	75	Nanshu 88	Nanchong Sichuan Academy of Agricultural Sciences
23	Chun'anhongxin	Chun'an County	76	Zhe 259	Zhejiang Academy of Agricultural Sciences
24	Zipibaixin	Jiande City	77	Zhezishu 4	Zhejiang Academy of Agricultural Sciences
25	Hongpibaixin-3	Chun'an County	78	Fanshu-1	Quzhou City
26	Shenglibaihao-3	Sanmen County	79	Hongpibaixin-9	Haiyan County
27	Nanguafanshu-1	Chun'an County	80	Zheshu 26	Zhejiang Academy of Agricultural Sciences
28	Taiwanfanshu	Cangnan County	81	Zhe 20	Zhejiang Academy of Agricultural Sciences
29	Beijingzi	Wuyi County	82	Huabei 18	Cangnan County
30	Hongpibaixin-4	Xinchang County	83	Yongtaishu	Cangnan County
31	Hongxinganshu	Jiashan County	84	Nanguafanshu-2	Chun'an County
32	Liushiri-2	Longquan City	85	Suxiang 4	Cangnan County
33	Guangsiwu	Sanmen County	86	Gao'erganshu	Pan'an County
34	Baifanshu	Yongkang City	87	Xinzhonghua-2	Cangnan County
35	Zhe 75	Zhejiang Academy of Agricultural Sciences	88	Hongpihuangxin-2	Ninghai County
36	Hongtou	Jinyun County	89	Pingguofanshu	Songyang County
37	Qingtengfanshu	Yongkang City	90	Xueshu	Zhejiang Academy of Agricultural Sciences
38	Zipihuangxin	Jiande City	91	Wanjinshu	Liandu County Lishui City
39	Hongpibaixin-5	Chun'an County	92	Ganshu	Pan'an County
40	Jinguafanshu	Liandu County Lishui City	93	Fanshu-2	Jiande City
41	Xiaoshanmudong	Xiaoshan District	94	71,438	Zhejiang Academy of Agricultural Sciences
42	Shiniuhongmudan	Liandu County Lishui City	95	Hongpibaixin-10	Shengzhou City
43	Zitong 2	Chun'an County	96	Wugecha	Jinyun County
44	Shenglibaihao-4	Chun'an County	97	Modong	Yuhuan County
45	Hongmudan	Cangnan County	98	Xushu 18–2	Xuzhou Sweet Potato Research Center
46	Yuguateng	Cangnan County	99	Hongpibaixin-11	Chun'an County
47	Rui'an	Xiaoshan District	100	Zhezishu 1	Zhejiang Academy of Agricultural Sciences
48	Baimahongxin	Chun'an County	101	Zhe 13	Zhejiang Academy of Agricultural Sciences
49	Xiaoyeqingteng	Jinyun County	102	Zhezishu 6	Zhejiang Academy of Agricultural Sciences
50	Mudanshu	Cangnan County	103	Zhecaishu 726	Zhejiang Academy of Agricultural Sciences
51	Liushiri-3	Suichang County	104	Zhe 21	Zhejiang Academy of Agricultural Sciences
52	Jinguahuang	Yongkang City	105	Taizhong 6	Qingdao agricultural Technology Extension Station
53	Baixinfanshu	Jiashan County			

### DNA fingerprinting and characteristics of RBIP markers

In the 64 bands of the 6 pairs of RBIP primers, 51 polymorphic bands were used to generate a DNA fingerprint map of the 105 sweet potato cultivars. For each primer pair, the number of loci ranged from 7 to 14 with an average of 10.7, while the number of polymorphic bands varied from 6 to 11 with an average of 8.5 (Table 3). The polymorphic bands were converted to digital fingerprint data with presence as “1” and absence as “0”. A “1”, “0” (Supplementary Table 2) digital fingerprint map was constructed by polymorphic loci. The digital fingerprint map was subsequently used to analyze the genetic diversity.

**Table 3:** Characteristics of the 6 RBIP primer pairs used for constructing sweet potato fingerprints.

Name	No. of alleles	No. of polymorphic alleles	Ne*	H*	I*	PIC
LTR10	7	6 (85.71%)	1.6464	0.3492	0.5111	0.2713
LTR11	10	9 (90.00%)	1.4024	0.2652	0.4237	0.2226
LTR13	12	9 (75.00%)	1.2206	0.1548	0.2761	0.1353
LTR20	9	6 (75.00%)	1.2534	0.1778	0.3064	0.1539
LTR37	12	10 (83.33%)	1.3808	0.2441	0.3931	0.2039
LTR38	14	11 (78.57%)	1.1512	0.1253	0.2369	0.1149
Mean	10.6666	8.5 (79.69%)	1.3231	0.2110	0.3479	0.1779

POPGENE software^26^ was used to further dissect the genetic variation among the 105 sweet potato cultivars using the 6 pairs of RBIP primers. The effective number of alleles (Ne*) ranged from 1.1512 to 1.6464, with an average of 1.3397. Nei’s gene diversity (H*) ranged from 0.1253 to 0.3492 among various genomic groups. The maximum gene diversity was in LTR10, followed by LTR11. Shannon’s index (I*) for each primer combination is also reported in Table 3. This index was highest in LTR10 (0.5111) but lowest in LTR38 (0.2369). To identify the most highly informative primer combination, the amount of polymorphism information content (PIC) was estimated from 0.1149 for LTR38 to 0.2713 for LTR10, with an average value of 0.1828 (Table 3).

### Genetic relationships among sweet potato accessions

**Figure 1:**
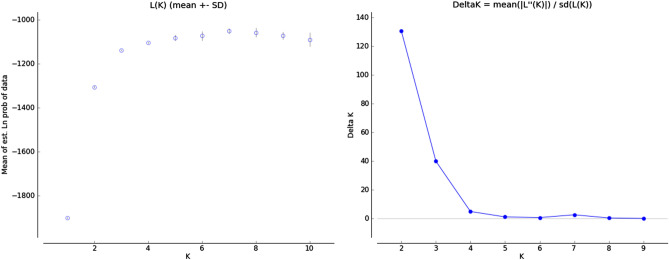
Modeling of cluster number for sweet potato using STRUCTURE. L(*K*) (left) and Delta *K* (right) were calculated in accordance with the method of Evanno et al.^28^.

**Figure 2:**
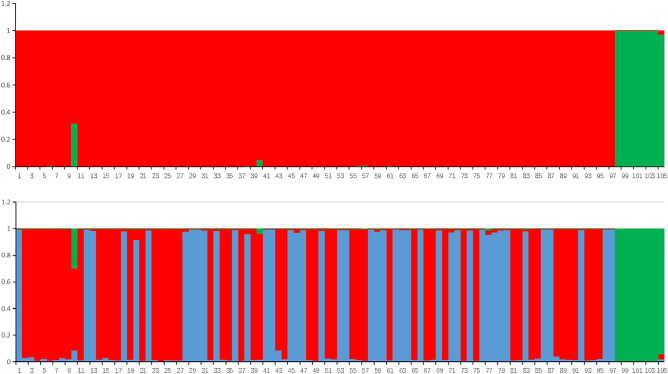
Genetic relationships among the 105 accessions of sweet potato revealed by a Bayesian modeling approach under *K* = 2 (top) and *K* = 3 (bottom) (numbers 1 to 105 represent the 105 sweet potato varieties).

Bayesian modeling of the number of homogeneous gene pools (*K*) in STRUCTURE^27^ was used to estimate the membership fractions of the 105 sweet potato accessions. An evaluation of the optimum value of *K* following the procedure described by Evanno et al.^28^ indicated two clear optimal values for Delta *K*, at *K* = 2 and 3 (Fig. 1), which indicated that a model with two gene pools captured a major split in the data and that substantial additional resolution was provided under a model with *K* = 3. Barplots of the proportional allocations to each gene pool for *K* = 2 and 3 were constructed in STRUCTURE and are shown in Fig. 2. The plots showed that these two models were related to each other hierarchically, such that the red cluster in the two-gene pool model was subdivided into two (blue and red) gene pools in the three-gene pool model.

The primary split in the data (*K* = 2) divided the accessions among two groups: group 1 and group 2. Group 1 (red in Fig. 2) included 97 sweet potato accessions, one of which was from America, while the remainder were from different provinces in China, and the majority of all the samples were from Zhejiang Province. Group 2 (blue) comprised 8 samples, six of which were from Zhejiang Province, and the remaining 2 were from Jiangsu and Shandong Provinces. The accessions that demonstrated a low level of admixture, except “Xushu 18-1”, belonged to *Ipomoea batatas* (L.) Lam. The model with 3 gene pools was also supported by the STRUCTURE results. Under this model, group 1 in the *K* = 2 model was further divided into two gene pools (red and blue), but the other gene pools remained almost the same (Fig. 2). The 3 groups included 53, 44, and 8 sweet potato accessions, respectively. In the *K* = 3 model, group 1 and group 2 (red and blue) overlapped substantially with one another, and the hierarchical levels in these two clusters could hardly be recognized. All 97 accessions in group 1 and group 2 appeared to be from the two major gene pools. Several accessions from Jiangsu Province showed admixed origins, such as ‘Xushu 18-1’, with three major gene pools.

**Figure 3:**
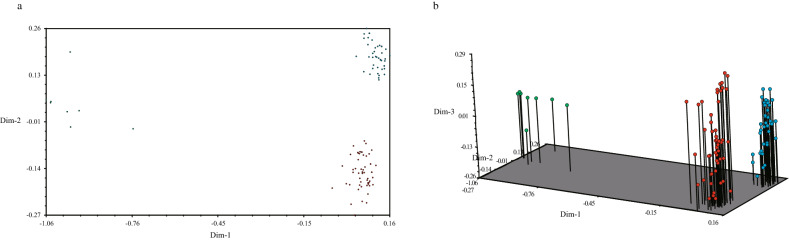
Two-dimensional (**a**) and three-dimensional PCAs (**b**) among the 105 sweet potato accessions based on 6 RBIP primer pairs (I represented by green, II by red, and III by blue).

A two-dimensional and three-dimensional PCA (principal component analysis) further depicted the relationship among the 105 sweet potato accessions (Fig. 3). In the two-dimensional PCA, Dim-1 and Dim-2 were 1.12 and 0.51, respectively. The Dim-3 was 0.60 in the three-dimensional PCA. From the PCA diagrams, we could see that all the 105 sweet potato accessions were divided into two groups, group 1 and group 2 (green and red, 8 and 97, respectively), or three smaller groups, group 1, group 2, and group 3 (green, red, and blue, 8, 53, 44, respectively). The PCA results were similar to the STRUCTURE results at *K* = 2, which classified all sweet potato accessions into two groups (red and blue, 97 and 8, respectively); however, in the *K* = 3 model, the red group was then divided into two groups (Fig. 2). The dimensionalities of the 3 groups indicated that the accessions in group 1 exhibited a higher genetic diversity than those in groups 2 and 3.

**Figure 4:**
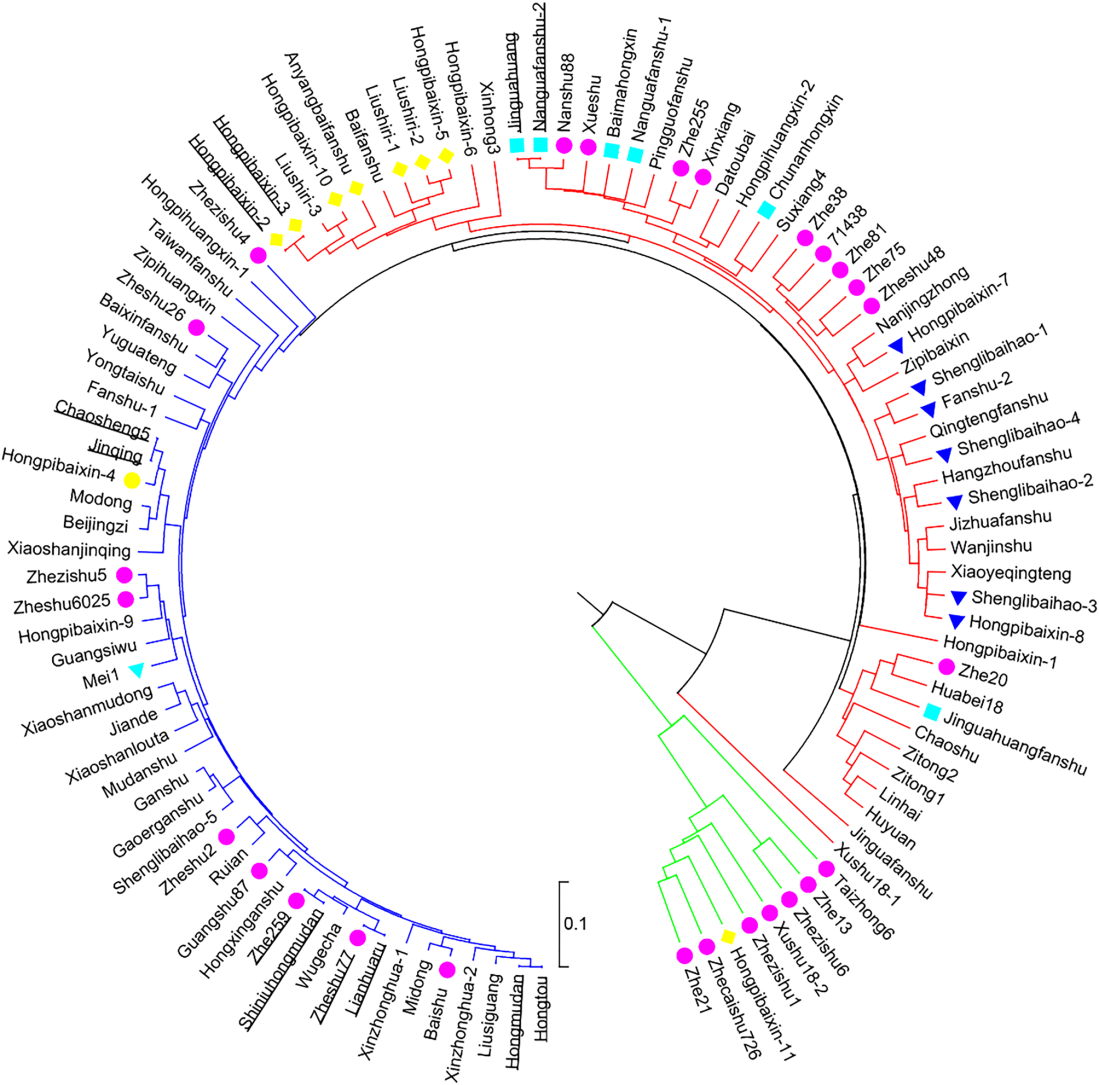
Dendrogram of 105 sweet potato accessions based on their genetic distances. UPGMA cluster analysis based on Dice’s similarity coefficients (Nei and Li, 1979) was used to generate the dendrogram. ‘Green branches’, ‘red branches’ and ‘blue branches’ represent the subbranches. Purple circles indicate improved varieties. Green triangles represent foreign varieties.

Neighbor-joining cluster analysis clearly divided the 105 sweet potato accessions into 3 groups containing 8, 54, and 43 materials, respectively. This result was highly consistent with the assignments made using STRUCTURE. (Fig. 4). Group 2 was divided into 4 subgroups, containing 11, 14, 5, 13, and 11 materials. Group 3 was divided into 5 subgroups, containing 9, 6, 5, 4, and 19 materials. Group 1 included all improved varieties, except Hongpibaixin-11, which had large genetic distances from the other accessions. For several accessions, such as ‘Jinguahuang’ and ‘nanguafanshu-2’ as well as ‘Hongpibaixin-2’ and ‘Hongpibaixin-3’, the genetic distances between them were 0, which meant that they had the same genotypes based on the 6 RBIP markers. The UPGMA dendrogram also showed that sweet potato accessions from the same regions were not well clustered in the same groups. For example, 22 accessions from the Zhejiang Academy of Agricultural Sciences were scattered. It was obvious that these results coincided with the previous STRUCTURE and PCA results.

A population differentiation analysis was performed to analyze the genetic variations among and within groups, as revealed by the population structure. In the *K* = 3 model, AMOVA revealed that 53% genetic differentiation (*P* < 0.001) of total molecular variance in the germplasm occurred among groups, and 47% (*P* < 0.001) was attributed to variation within groups (Table 4). The total genetic variance among individuals within populations was significantly greater than 0 (0.526), indicating that the genetic variation between and within the geographical population of the tested sweet potato resources was extremely significant (Table 4). Genetic distance among the 3 inferred groups revealed that Group 2 (blue) and Group 3 (green) had the highest differentiation with 0.819, and comparatively, Group 1 and Group 2 had a closer relationship with 0.265. The pairwise fixation index (*F*_ST_) values between the three populations were all 0.001 (Table 5).

**Table 4:** AMOVA results of 105 sweet potato germplasms with the *K* = 3 model.

Source	df	Sum of squares	Mean of squares	Estimated Variance	Percentage of total Variance (%)	AMOVA statistics	Value	P
Among Groups	2	225.601	112.800	3.698	53			
Within Groups	102	339.371	3.327	3.327	47	PhiPT^4^	0.526	0.001
Total	104	564.971		7.026	100

**Table 5:** Genetic distance (down diagonal) and pair fixation index (*F*_ST_, up diagonal) between three groups inferred by structure analysis.

Group	Group_1	Group_2	Group_3
Group_1		0.001	0.001
Group_2	0.265		0.001
Group_3	0.727	0.819	

## Discussion

To support sweet potato breeding programs, it is essential to assess the genetic diversity and relationships among cultivars. The ubiquity and abundance of LTR retrotransposons in the plant genome have made them valuable for studying genome-wide variation and diversity. The retrotransposon-based genetic DNA fingerprinting method could provide potentially useful genetic information. The increasing amount of sequence data released by next-generation sequencing technology provides a valuable resource for the development of retrotransposon-based markers. These retrotransposon-based markers have been applied successfully to analyze the genetic diversity in various plant species.

In the current study, we confirmed that the LTRs of sweet potato accessions contained the full complement of LTR retrotransposons. Structural analysis revealed that they are transcriptionally active and could be functional. Based on this genome-wide analysis, we found that only 12.5% of RBIP markers generated polymorphic bands, signifying that inter-LTR regions in the research genome of sweet potato accessions were significantly conserved. This implies that the sweet potato genome is still under evolution and that LTRs are not very active in contributing to genome-wide variations. To the best of our knowledge, this is the first study of genetic diversity in sweet potato using RBIP-based fingerprinting.

A previous report indicated that 7.37% of the sweet potato genome (approximately 4.4 Gb) was identified as an LTR^29^, while it was 10.987% in the present study. This small difference in the number of LTRs might be attributed to the different approaches and parameters that were used in the two studies. In our study, only putative full-length LTR retrotransposons with two very similar LTR sequences were isolated. The ratio of *Ty3-gypsy* to *Ty1-copia* can reflect the contribution in the sweet potato genome. Our results showed that full-length *copia* LTR retrotransposons were more common than *gypsy* retrotransposons (Table 1). The ratio of *Ty3-gypsy* to *Ty1-copia* was (1:1.6), higher than previous study (1:1.15)^29^. The numbers of full-length LTR retrotransposons in the different subfamilies were generally low, *gypsy* subfamilies had more single sequences (95.8%) than *copia* subfamilies (94.9%), and only 0.6% (49) contained more than 3 LTR retrotransposons. These findings are consistent with the results reported in other plants with different genome sizes^30–32^.

New bioinformatics software offers exciting perspectives for the development of new markers based on whole genome sequences. RBIP markers were more ubiquitous than SSR markers in sweet potato^29^. Although many SSR markers have been developed from sweet potato, almost all SSRs (86.1%) have mononucleotide or dinucleotide repeat motifs, and “stutter bands” or increased mutation rates in repeat lengths may create issues for using SSR markers^33–35^. RBIP markers amplify a single locus in samples, differing from SSR markers that potentially amplify two or possibly more homologous loci. RBIP markers also can detect the presence or absence of retrotransposons in a locus produced by the integration of an element^36^.

In our 48 developed RBIP markers, 21 and 27 pairs of primers were related to the insertion of *copia* and *gypsy* retrotransposons in a particular locus, respectively. Due to the high similarity of LTRs from the same subfamily, primers designed with these LTRs may produce a same left primer sequence. For example, the left primer sequences of LTR10, LTR11, LTR13 and LTR20 were the same, but the downstream sequences were different. This kind of situation does not influence the specific amplification. The insertion of *copia* and *gypsy* retrotransposons was extensively detected in most of the cultivars with more than one locus. Diversity analysis showed that *copia* and *gypsy* LTR retrotransposons have existed in sweet potato varieties for a long time. These results implied that *copia* and *gypsy* retrotransposons replicated many times in the development of cultivated sweet potato and might explain why 10.98% of the genome was LTR retrotransposon in this research. Additionally, compared with previous RBIP markers^1^, the genome based RBIP markers have universal applicability.

According to the STRUCTURE analysis, the 105 germplasms can be divided into two groups when *K* = 2 in STRUCTURE (Fig. 2). Almost all the germplasms had unique backgrounds, except ‘Xushu18-1’, ‘Jinguafanshu’, and ‘Taizhong6’. ‘Xushu 18-1’ (p330683034) was released by the Xuzhou Regional Agricultural Research Institute in 1972. It was selected from the cross between ‘Xindazi’ X ‘52-45’ with an inbreeding backcross, and ‘52-45’ was a hybrid offspring of ‘Nancy Hall’ X ‘Okinawa 100’. Previous studies have shown that most of the sweet potato cultivars have a genetic background of Okinawa 100 from Japan and Nancy Hall from the USA^7,10,37,38^. Based on the above research, we inferred that the two gene pools may be Nancy Hall and Okinawa 100. However, in the *K* = 3 model (Fig. 2), most of the accessions had one major gene pool source and a small minor gene pool, except ‘Xushu 18-1’. From these results, we can see that ‘Xushu 18-1’ has a wider genetic background than other accessions. The genetic background of sweet potato was single in Zhejiang, even among China, so it is necessary to broaden the genetic background of sweet potato varieties, enrich their genetic diversity and protect high-quality germplasm resources.

All the accessions can be divided into three groups (group I represented by green, group II by red, and group III by blue) according to the PCA results and UPGMA dendrogram (Figs. 3, 4). In group I, 7 of the 8 accessions were improved varieties, and the other 21 improved varieties were scattered in subgroups II and III. This result indicated that the genetic background of improved varieties had more similarity than other germplasms to some degree. ‘Hongpibaixin-11’ is a variety selected by local farmers and known for its phenotypic traits such as leaf shape, root tuber skin color, and root tuber flesh color. There were 11 and 9 improved varieties in groups II and III, respectively, and other landraces were scattered in the two groups. The varieties ‘Zhe 38’, ‘71438’, ‘Zhe 81’, ‘Zhe75’, and ‘Zheshu 48’, which were improved by the Institute of Crop and Nuclear Technology Utilization, Zhejiang Academy of Agricultural Sciences, clustered on the third subgroup of group I, illustrating that these varieties had similar genetic backgrounds in the hybrid combination. ‘Zhe 255’ and ‘Xinxiang’, ‘Zhezishu 5’, ‘Zheshu 6025’ and ‘Nanshu 88’ were the same.

The UPGMA dendrogram, PCA, and STRUCTURE analysis remained highly consistent regardless of *K* = 2 or *K* = 3 (Figs. 2, 3, 4). The PCA results showing that the sweet potato germplasms in the three groups were very concentrated, and the genetic differences of the three groups were obvious. The genetic distance between Group_1 and Group_2 was 0.265, that between Group_1 and Group_3 was 0.727, and that between Group_2 and Group_3 was 0.819 (Table 5). The above data indicated that the accessions between Group_2 and Group_3 had the widest genetic background, followed by accessions between Group_1 and Group_3, and between Group_1 and Group_2. The pairwise fixation index (*F*_ST_), as a population differentiation index determined by genetic structure, can often be used to assess genome-wide variation. The mean *F*_ST_ value between the three groups were 0.001, indicating that there was a very high level of differentiation between the three groups. Thus, the germplasms in the three groups could be combined as good hybrid parents.

AMOVA showed that the source of variation among and within populations was 53% and 47%, respectively, indicating that the genetic variance was significant in the tested resources (Table 4). Most of the sweet potato resources have been produced in Zhejiang Province for a long time, and local environmental conditions have a significant effect on genetic variation. This result was inconsistent with those of previous studies^7,9^. The proportion of the total genetic variance among individuals within populations (PhiPT) value was 0.526, and *P*<=0.001, showing that the total genetic variance among individuals within populations was extremely significant.

We found that several germplasms with similar phenotypes were separated into close subgroups. For example, ‘Hongpibaixin-7’, ‘Shenglibaihao-1’, ‘Fanshu-2’, ‘Shenglibaihao-4’, ‘shenglibaihao-2’, ‘shenglibaihao-3’, and ‘Hongpibaixin-8’, with similar phenotypic characters of leaf shape, leaf teeth type, leaf color, stem primary color, root tuber skin color, and root tuber flesh color (Supplementary Table 3), clustered together in the second subgroup of the second group; The germplasms ‘Hongpibaixin-4’, ‘Hongpibaixin-2’, ‘Hongpibaixin-3’, ‘Hongpibaixin-10’, ‘An’yangbaifanshu’, ‘Liushiri-1’, ‘Liushiri-2’, ‘Hongpibaixin-5’ and ‘Hongpibaixin-11’, with similar phenotypic characters clustered in the fifth subgroup of the second group. This phenomenon may be attributed to most of the germplasms collected from locals being used for planting for many years. Long-term self-retention may cause a germplasm resource to exhibit segregation of variables. The UPGMA genetic relationship reflects the difference in genetic background between germplasm resources, so selection of genetically distant accessions as hybrid parents in breeding is more likely to generate elite varieties. Our results have demonstrated the high potential of molecular marker-based parental selection in promoting genetic improvement in future sweet potato breeding programs.

However, several germplasm resources, such as ‘Shenglibaihao-1’, ‘Shenglibaihao-2’, ‘Shenglibaihao-3’, and ‘Shenglibaihao-4’, collected from different counties and cities of Zhejiang Province, called the same name (Shenglibaihao) by local people, were not similar in terms of the phenotypic characters of leaf vein color and leaf stalk color. Thus, several germplasm resources were numbered and considered synonymous. From the results of the UPGMA dendrogram, however, we could see that those synonymous germplasm resources were not clustered together. They were not a same variety. ‘Shenglibaihao’, also named Okinawa 100, was bred in Japan and then introduced to China before the 1970s. Almost 90% of the genetic background of improved varieties in the 1960s was filial generations of ‘Shenglibaihao’^7,10,37,38^. The filial generations that have phenotypic traits similar to those of ‘Shenglibaihao’ may also be called ‘Shenglibaihao’ by farmer breeders, which could be the reason why there were resources named ‘Shenglibaihao-1’, ‘Shenglibaihao-2’, ‘Shenglibaihao-3’, and ‘Shenglibaihao-4’, with different variations but clustered together. The synonymous landraces ‘Hongpibaixin’, ‘Liushiri’, and ‘Fanshu’ have similar situations. The landraces ‘Hongpibaixin-2’ and ‘Hongpibaixin-3’, collected from Cangnan County, Wenzhou City, and Jinyun County, Lishui City, Zhejiang Province, China, respectively, showed 100% similarity in the UPGMA dendrogram, STRUCTURE, and PCA results. The 6 RBIP primer pairs used in this study amplified the same fragments in these two accessions (Supplementary Table 2), so we speculated that these two accessions might be synonyms. Investigation of phenotypic traits (Supplementary Table 3) confirmed our speculation. The same situations existed between ‘Jinguahuang’ and ‘Nanguafanshu-2’ as well as ‘Zheshu 77’ and ‘Lianhuaru’. However, we did not observe the same phenotypic characteristics between ‘Hongmudan’ and ‘Hongtou’, ‘Chaosheng 5’ and ‘Jinqing’, ‘Zhe 259’ and ‘Shiniuhongmudan’. The reason may be that these sweet potato germplasm resources had very similar genetic backgrounds, and more markers will be needed to confirm their relationships.

The results in this research expanded the application of molecular markers in sweet potato. Successfully developing RBIP markers, evaluating the capacity and efficiency of 6 RBIP markers for distinguishing genetic diversity in 105 germplasm resources of Zhejiang Province. The clustering results combined with phenotypic characteristics were used to identify several germplasm resources. The importance of molecular markers in variety identification was further confirmed. This is the first RBIP-based and combined with phenotypic characteristics genetic diversity assessment in sweet potato. These results will play a great role in sweet potato genetic research and breeding programs.

## Conclusions

In this study, we successfully developed 48 RBIP primer pairs from the sweet potato genome, and successfully analyzed the genetic diversity and constructed a fingerprint of 105 sweet potato germplasm resources based on 6 RBIP primer pairs. These sweet potato germplasm resources exhibited a relative narrow genetic background due to scarce backbone parents and geographical isolation. This study highlights the utility of RBIP markers for determining the intraspecies variability of sweet potato. These highly polymorphic RBIP primer pairs have the potential to be used as core primer pairs for variety identification, genetic diversity assessment and linkage map construction in sweet potato. All these findings could provide a good theoretical reference and guidance for germplasm research and breeding.

## Materials and methods

### Plant materials and DNA extraction

All 105 sweet potato cultivars used in the present study are donated by the Sweet potato Germplasm Repository, the Institute of Crop and Nuclear Technology Utilization, Zhejiang Academy of Agricultural Sciences, Hangzhou, China (Table 2). They collected these resources from Zhejiang Province according to the <Implementation Plan of the Third National Crop Germplasm Resources Survey and Collection Action> issued by the Ministry of Agriculture and Rural Affairs of China.The samples included 26 improved varieties, 78 landraces from different geographical regions and 1 introduced variety from the United States of America. Total genomic DNA was extracted from fresh young leaves with the modified cetyltrimethylammonium bromide (CTAB) method described by Saghai-Maroof et al.^39^ The quality and quantity of DNA were detected using spectrophotometric analyses and 1% (w/v) agarose gel electrophoresis, respectively. The DNA was diluted to a final concentration of 50 ng/μL and then stored at − 80 °C for further use. The phenotypic traits of the cultivars were also investigated for their genetic information assessment, including leaf shape, leaf tooth type, top bud color, tip hair color, leaf vein color, petiole color, stem primary color, stem secondary color, root tuber shape, root tuber skin color, and root tuber flesh color (Supplementary Table 3).

### LTR-RT prediction in the sweet potato genome

The full-length sequences of LTRs were predicted using LTR harvest software based on the genomic data of sweet potato variety ‘Taizhong 6’, which was downloaded from the website http://public-genomes-ngs.molgen.mpg.de/Sweetpotato/. The parameters of LTR harvest were set as follows: (1) the length range of the LTRs was 100–1000 bp; (2) the distance between the starting points of the LTRs was 1000-15000 bp; (3) the similarity threshold was 85%; (4) the repeat sequence length of each target site was 4–20 bp; (5) there were 4–6 bp target site duplications (TSDs) or polypurine tracts (PPTs) and primer binding sites (PBSs) on both sides of two identical LTRs; and (6) the other software parameters were set to the default options. The sequences of predicted LTRs were translated into six codes to obtain the corresponding protein sequences. All the *copia* and *gypsy* gene models were downloaded from the PFAM tadabase (gag, pf03732; integrate, pf00665; reverse transcriptase, pf00078 and pf07727) (http://pfam.xfam.org/), and an HMM (*gag*, *INT*, *RT*) was constructed based on the downloaded data. The functional domain sequences of LTR protein sequences were subsequently analyzed using BLASTN searches (E-value< 1e−10) against the HMMs. By searching the three models in each protein sequence, LTRs containing at least two models were screened for subsequent analysis. The screening criteria were a full-length E-value < 1e−10 and an optimal domain E-value< 1e−10.

### Development and evaluation of RBIP primers

The RBIP primers were designed by Primer3^40^. One primer was designed from LTR sequence and another was designed from flanking genome sequence. The design principles were as follows: (1) the primer length was 18–25 bp; (2) the amplified products were 100–1000 bp; (3) the GC content of the primers was 35–55%; (4) the annealing temperature was 50–60 °C; and (5) the annealing temperature difference between upstream and downstream primers was less than 5 °C. The designed primers were synthesized by Beijing Tsingke Biotechnology Co., Ltd.

PCR amplification was carried out in 15 μL reaction solution consisting of 1 μL DNA template, 7.5 μL Tsingke Master Mix (Tsingke, Beijing, China), 1 μL (10 μmol L–1) of each RBIP primer (Tsingke, Beijing, China) and 4.5 μL deionized distilled water. PCR amplification was performed with the following procedure: 94 °C for 5 min followed by 35 cycles at 94 °C for 30 s, 58–60 °C (depending on the RBIP primers) for 30 s, 72 °C for 30 s, a final extension at 72 °C for 5 min, and storage at 4 °C. Amplicons were analyzed by electrophoresis on a 2% (w/v) agarose gel. Amplicons were pooled together with an internal size standard (ABI GeneScanTM 500 LIZ, Applied Biosystems, Foster City, USA) and loaded on an ABI Genetic Analyzer (3730XL, Applied Biosystems, Foster City, USA). Accurate allele points were analyzed by Gene mapper 4.1 software^41^.

### Data analysis

Characteristics of the RBIP primer pairs developed for analyzing sweet potato genetic diversity were evaluated in the 105 sweet potato accessions in terms of the effective number of alleles (Ne*), Nei’s gene diversity (H*), Shannon’s information index (I*) and polymorphism information content (PIC) using POPGENE version 1.32^26^ and the Botstein formula^42^, respectively.

For nonhierarchical genotypic clustering, the number of homogeneous gene pools (*K*) was modeled using the genotypes obtained from all 105 individuals in the software STRUCTURE version 2.3.3, which uses the Markov chain Monte Carlo (MCMC) algorithm^43,44^. This revealed the genetic structure by assigning individuals or predefined groups to *K* clusters. Twenty runs of STRUCTURE were performed by setting the number of clusters (*K*) from 2 to 10. Each run consisted of a burn-in period of 100,000 iterations followed by 100,000 MCMC iterations, assuming an admixture model. The results were uploaded to the STRUCTURE HARVESTER website (http://taylor0.biology.ucla.edu/STRuctureHarvester/^27^) to estimate the most appropriate *K* values. The replicate cluster analysis of the same data resulted in several distinct outcomes for estimated assignment coefficients, even though the same starting conditions were used. Therefore, CLUMPP software was employed to average the 20 independent simulations, and the results were illustrated graphically using DISTRUCT^45^.

All the “1” and “0” data were used to calculate Dice’s similarity coefficients and genetic distances^46^ among the 105 sweet potato accessions by the NTSYS-pc version 2.2 statistical package^47^. A UPGMA dendrogram based on the genetic distance matrix was constructed by MEGA X software^48^ to evaluate genetic relationships among the sweet potato varieties. Two-dimensional and three-dimensional PCAs were constructed with the R statistical package^49^ and used to indicate the distribution of individual varieties in the scatter diagram.

To investigate the genetic differentiation among the 105 sweet potato accessions, AMOVA was performed based on population inference according to structure analysis by the software Arlequin v3.5^50^, with 1,000 permutations and sum of square size differences as molecular distance. Furthermore, pairwise differentiation levels were estimated by the pairwise *F*_ST_, a measure of heterozygosity among populations relative to heterozygosity within populations^51^.

## Supplementary Information

Supplementary Figure 1.

Supplementary Table 1.

Supplementary Table 2.

Supplementary Table 3.
