# Supplementary material for: Assessment of genetic diversity and variety identification based on developed retrotransposon-based insertion polymorphism (RBIP) markers in sweet potato (Ipomoea batatas (L.) Lam.)
Source: Sci Rep. 2021 Aug 24;11:17116. doi: 10.1038/s41598-021-95876-w (PMC8385064; doi:10.1038/s41598-021-95876-w)
Supplement: Supplementary file 1 — Supplementary Figure 1. [file 41598_2021_95876_MOESM1_ESM.docx]

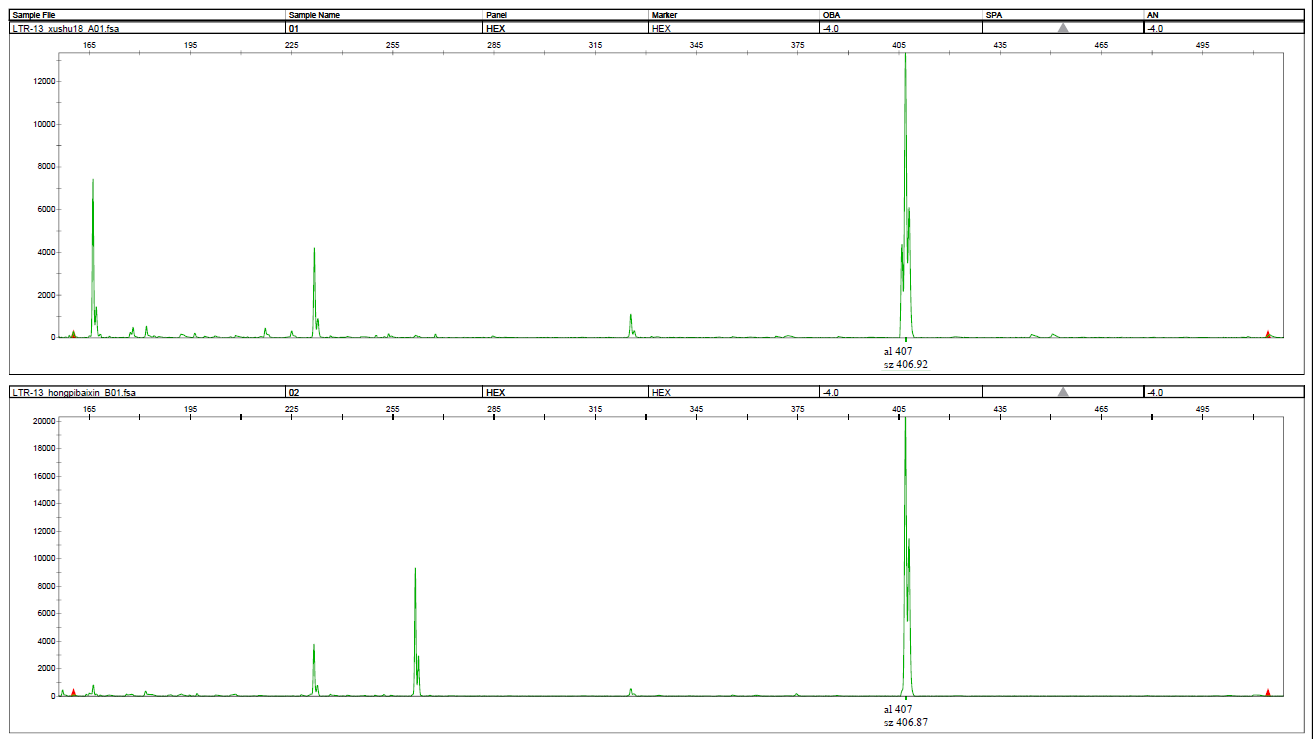


Supplementary Figure 1 locus information on 2 sweetpotato accessions amplified by LTR-13 primer pair.
